# Development of a potential nano-based delivery system combining Colchicine-loaded lipid nanocapsules and BIOT-NFL-peptide to target glioblastoma

**DOI:** 10.1016/j.ijpx.2025.100382

**Published:** 2025-08-26

**Authors:** H. Alnemeh-Al Ali, J. Bejaud, N. Lautram, A. Dupont, J. Eyer

**Affiliations:** a Univ Angers, Inserm, CNRS, MINT, SFR ICAT, F-49000 Angers, France; b Univ Rennes, CNRS, Inserm, BIOSIT-UMS 3480, US_S 018 Rennes, France

**Keywords:** Targeted delivery, BIOT-NFL-peptide, Colchicine-loaded lipid nanocapsules, Nanosystem (nanofibers/nanocapsules), Glioblastoma

## Abstract

Peptide conjugated-nanodrug is one of the most studied new treatment options in the field of cancer, including glioblastoma (GBM). This tumour, GBM, is a difficult to treat brain tumour mainly due to its location and the complexity of targeting tumour cells. A promising GBM-targeting peptide (NFL-TBS.40–63) is a cell penetrating peptide (CPP) that has previously demonstrated selectivity against GBM cells and also a capacity to damage their microtubule network at appropriate concentrations. Here, a potential nano-based delivery system was developed by coupling the biotinylated-NFL-peptide (BIOT-NFL) with lipid nanocapsules (LNCs) loaded with Colchicine (Col), an anti-microtubule agent with potent anticancer activity. The effect of BIOT-NFL and free Colchicine was first evaluated on GBM cells. Colchicine was then loaded in lipid nanocapsules (Col-loaded LNCs) and the obtained nanocapsules were characterized for particle size, morphology, encapsulation efficiency and *in vitro* release, and then coupled with the BIOT-NFL-peptide. Interestingly, a potential nanosystem composed of peptide-nanofibers (formed from BIOT-NFL) decorated with Col-LNCs was observed by electron microscopic examination. Finally, the effects of this BIOT-NFL coupled to Col-LNCs were evaluated *in vitro* on GBM cells. This new nanosystem may offer a promising strategy for GBM targeted therapy.

## Graphical abstract

**Figure:**
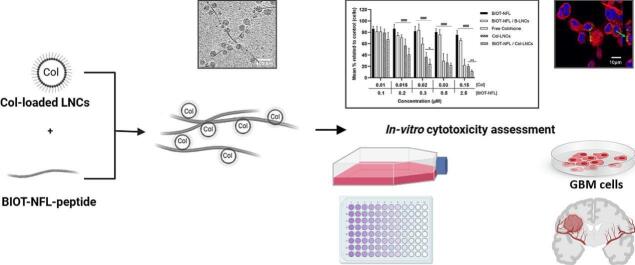

## 1 Introduction

Glioblastoma (GBM) is one of the deadliest brain cancers characterized by a very poor patient prognosis, even with the best treatments available nowadays (neurosurgery, radiotherapy, and chemotherapy) (Thakkar et al., 2014; Hanif et al., 2017). This tumour is accompanied with a range of complications related to the underlying disease as well as several challenges like the presence of the blood-brain barrier, the lack of specificity of therapeutics, and also the development of drug resistance mechanisms (Ganipineni et al., 2018), that altogether cause the failure of actual treatments. Drug delivery is another major challenge in the context of GBM due to the complexity of targeting tumour cells located mainly in the supratentorial region of the brain (Tamimi and Juweid, 2017). Due to all these limitations, there is an urgent need to develop new therapeutic strategies to combat GBM.

To develop new effective therapies, many research groups have focused on nanomedicines to fight this cancer (Alphandéry, 2020). Novel delivery systems based on nanostructures (nano-based delivery systems) are emerging as promising choices for improving delivery and targeting of cancer cells. Nano-based drug delivery systems also permit the combination of anti-cancer drugs, in a way that synergistically or additively targets them into cancer cells (Zhao et al., 2020). Over the past decades, nano-therapies have been widely studied and have shown great promise in the treatment of cancer, including GBM (Zhao et al., 2020). Respective efforts to develop a novel delivery system based on nanostructures that can protect the drug from degradation, bypass the BBB, and that are specific to tumour cells are ongoing. Nevertheless, the delivery of drugs to the tumour site remains a major challenge in the case of GBM. Therefore, smart structural design is still needed to improve the targeting of glioblastoma nano-treatments (Béduneau et al., 2007).

NFL-TBS.40–63 (NFL-peptide), corresponding to the sequence of a tubulin-binding site on neurofilaments, is a promising GBM-targeting peptide that has previously shown its selectivity against GBM cells without damaging normal brain cells (Berges et al., 2012a). NFL-peptide acts as a cell-penetrating peptide (CPP) that can specifically penetrate GBM cells *via* endocytosis pathway (Lépinoux-Chambaud and Eyer, 2013). This peptide also revealed its anti-tumour activity in GBM cells, at appropriate concentrations, where it destroys their microtubule network and induces cell death by interacting with unpolymerized free tubulin and inhibiting microtubule polymerization (Berges et al., 2012b).

Recently, some of us demonstrated the ability of this Biotinylated-NFL-peptide (BIOT-NFL) to form peptide-nanofibers in several biological solutions at physiological pH. The nanofibers obtained from the BIOT-NFL-peptide (with a diameter of approximately 5 nm in width and several micrometers in length) have been investigated as tumour-targeting nanofibers for the delivery of nanoparticles into GBM cells (Griveau et al., 2022). Interestingly, these nanofibers showed an important interaction with other nanoparticles, like gold nanoparticles, with an enhancement of particles uptake by GBM cells when treated with this system (BIOT-NFL-nanofibers/gold nanoparticles), compared to gold nanoparticles alone (Alnemeh-Al Ali et al., 2022). Finally, when the liposomes are functionalized with this BIOT-NFL peptide, it has been shown in the laboratory using an *in-vitro* model of blood-brain barrier passage that the liposomes are capable of crossing this barrier (Mellinger et al., 2023). This is currently being confirmed *in vivo* (data not shown). All these findings have inspired the use of the BIOT-NFL peptide as a GBM-targeting vector combined with other lipid nanocapsules loaded with an anti-cancer drug to target glioblastoma.

Colchicine (Col) is a high potential anticancer drug that binds the tubulin at Colchicine binding site (CBS) and inhibits microtubule polymerization inducing cell death (Naaz et al., 2019). Thus, colchicine acts similarly to the NFL peptide. Agents that disrupt the structure or function of microtubules have been shown to be particularly toxic to cancer cells in preclinical and clinical settings (Kumar et al., 2017). Colchicine was the first identified tubulin destabilizing agent and is commonly used as an FDA approved drug (since 2009) to treat several diseases like gout, familial Mediterranean fever and Behçet's disease. Colchicine and other CBSIs (Colchicine binding site inhibitors) have also been widely explored in preclinical studies as potential anti-cancer drugs and they remarkably proved their activity against several cell lines *in vitro* as well as *in vivo* models (Fakih et al., 1995; Lin et al., 2013). Furthermore, several Colchicine derivatives have shown to be effective in drug-resistant models comparing to other anti-microtubules classes, like Taxane or Vinca alkaloid, whose effectiveness has been limited by the development of resistance mechanisms (Arnst et al., 2019; Yang et al., 2018). Hence, many efforts have continuously been made to develop colchicine or its derivatives as anti-cancer drugs because of their favourable factors; simple structure, easy to synthesize, high antitumor potent and low cost (Wu et al., 2015; Yang et al., 2018).

Previous studies have reported that very low doses of this compound (Col) can reduce cancer cell proliferation and decrease tumour size in animals following intravenous administration (Wu et al., 2015). In glioma models, a Colchicine derivative named AD1 successfully inhibited cell proliferation of human glioblastoma cell lines (U87MG and U373MG) at nanomolar scale doses, and suppressed the tumour growth when injected in rat bearing glioma (Fang et al., 2015). Despite the reported non-specific toxicity of Colchicine to healthy cells, low concentrations of this compound have been shown to be clinically acceptable for the treatment of cancer (Lin et al., 2013). Here, the Colchicine was selected to be loaded into lipid nanocapsules and coupled with the BIOT-NFL-peptide to be administered in glioma models.

Lipid nanocapsules (LNCs) are solvent-free nanocarriers that have been established in our laboratory in 2002 to encapsulate anti-cancer drugs and overcome their clinical limitations, like high toxicity and lack of specificity (Heurtault et al., 2002). Several anticancer drugs have been successfully loaded into these LNCs and have shown improvement in drug bioavailability and efficacy when using *in vitro* and *in vivo* models (Briot et al., 2017). Besides, LNCs can be surface functionalized with various targeting ligands such as antibodies, proteins, and peptides, which in turn can be employed to guide these vehicles to the target site (Béduneau et al., 2007; Lollo et al., 2015).

Based on the GBM-targeting effect of BIOT-NFL-peptide as well as its other profits, our goal in this study was to establish a novel nanosystem that may combine BIOT-NFL-nanofibers with lipid nanocapsules (LNCs) loaded with the anti-microtubule agent (Colchicine). In this context, our work aimed to i) assess the toxic effect of BIOT-NFL combined with Colchicine on rat F98 glioblastoma cells, ii) elaborate lipid nanocapsules loaded with Colchicine (Col-LNCs), iii) study the interaction between BIOT-NFL-nanofibers and Col-LNCs using electron microscopy examinations, and finally iv) investigate *in-vitro* the cytotoxic effect of Col-LNCs coupled with BIOT-NFL on GBM cells. Owing to BIOT-NFL-peptide features, this nanosystem (BIOT-NFL-nanofibers/Col-LNCs) is expected to be a potential nano-based delivery system to target GBM.

## 2 Materials and methods

### 2.1 Chemicals

NFL-TBS.40–63 peptide (NFL-peptide) coupled to the biotin with a molecular mass of 2.7 kDa was synthesized by PolyPeptide group (Strasbourg, France). The powder of biotinylated-NFL-TBS.40–63 peptide (BIOT-NFL) was dissolved in Milli-Q purified water before use. Colchicine (MW = 399.44 g/mol) was supplied by Sigma-Aldrich (C9754; India). A stock solution of Colchicine was prepared by dissolving the Colchicine in ethanol (absolute ethanol; Fisher Scientific, UK) at a concentration of 50 mg/mL. This solution was stored at 4 °C in the dark and brought to room temperature before use.

For LNCs preparation, the lipophilic Labrafac WL 1349 (caprylic-capric acid triglycerides) was provided by Gatefossé SA. (Saint-Priest, France). Lipoïd S100 (soybean lecithin at 69 % of phosphatidylcholine) was obtained from Lipoïd Gmbh (Ludwigshafen, Germany) and Kolliphor HS15 (mixture of free polyethylene glycol 660 and polyethylene glycol 660 hydroxy stearate) was obtained from BASF (Ludwigshafen, Germany). NaCl was purchased from VWR Chemicals (Belgium). Deionized water was obtained from a Milli-Q plus® system (Millipore, Billerica, USA).

### 2.2 Cell culture

Rat F98 glioma cells (obtained from ATCC, USA) were cultured in T75 flasks in Dulbecco's Modified Eagle Medium-high glucose (Sigma-Aldrich), supplemented with 10 % fetal bovine serum (Sigma-Aldrich), 1 % non-essential amino acids (Sigma-Aldrich), and 1 % of 100× penicillin/streptomycin (Sigma-Aldrich, Israel). Cells were washed with 1× DPBS (Dulbecco's Phosphate Buffered Saline; Sigma-Aldrich). Trypsin solution (0.25 %) from Sigma-Aldrich was used for cell dissociation. The cells were maintained in a humidified incubator with an atmosphere containing 5 % CO_2_ at 37 °C and passaged every 2–3 days. Human pancreatic cancer (MIA PaCa-2) and human neuroblastoma (SH-SY5Y) cell lines were cultured under the same conditions and also used for the cytotoxicity MTT assay.

### 2.3 Analytical method of Colchicine (UPLC-UV)

Ultra-performance liquid chromatography (UPLC) with UV detection was performed using an Acquity® H-Class Bio UPLC apparatus with Acquity UPLC® BEH C18 column (50 mm × 2.1 mm, 1.7 μm particle size) provided from Waters. The analysis of Colchicine was carried out by using a gradient elution method with 0.15 % phosphoric acid (A) and acetonitrile (B) as mobile phase (flowrate 0.1 mL/min). The gradient profile started with 90 % mobile phase A and reached 100 % mobile phase B in 2 min according to a linear curve. This condition was maintained for 8 min, then the system returned to the initial condition, switching back to 90 % mobile phase A at 10.5 min and lasting until 15 min. Wavelength of the UV detector was set at 350 nm (Chen et al., 2007). Colchicine solutions were prepared in the absolute ethanol (HPLC grade) obtained from Fisher Scientific. A symmetrical peak of Colchicine was detected with a retention time of 5.1 min (Fig. S1). A calibration curve of Colchicine dissolved in ethanol was obtained by quantifying the area under the peak (coefficient of determination R^2^ = 0.997). All samples containing Colchicine were analyzed using this method, and the area under the peak was proportional to Colchicine concentration.

### 2.4 Analysis of the effect of BIOT-NFL-peptide and free Colchicine on GBM cells

#### 2.4.1 MTT assay

Rat F98 glioma cells were seeded in a 96-well flat-bottom microtiter plate at a density of 1 × 10^3^ cells/well and allowed to adhere for 24 h at 37 °C in a CO_2_ incubator. To evaluate the effect of the two agents (BIOT-NFL and Colchicine), cells were treated with free BIOT-NFL, free Colchicine, and BIOT-NFL combined with Colchicine following the removal of culture media. After 72 h of treatment, 10 μL of MTT working solution [3-(4,5-dimethyl thiazolyl-2)-2,5-diphenyltetrazolium bromide obtained from Invitrogen, USA] prepared at 5 mg/mL in phosphate buffer solution, was added to each well, and plates were subsequently incubated at 37 °C for 3 h. After the culture supernatants were removed, 150 μL DMSO was added to each well, and the absorbance was measured at a 570 nm wavelength. Three parallel wells were performed in each experiment. The absorbance of cells with only the culture media (not treated) was considered as 100 %. Experiments were performed at least in triplicate.

#### 2.4.2 Tubulin immunofluorescence

30,000 cells of rat F98 glioma per well were cultured on coverslips in a 24-well plate and incubated at 37 °C in a CO_2_ incubator. After 48 h, the medium was discarded, and adherent cells were treated with BIOT-NFL with or without the Colchicine for 6 h. A control of cells without treatment has always been done. Following treatment, cells were washed with 1× PBS (Gibco) and fixed for 10 min in 4 % paraformaldehyde (15714S; Electron Microscopy Science, England). They were then incubated in a permeabilization solution 0.2 % Triton X-100 (T9284; Sigma-Aldrich) in PBS for 10 min before incubation in 5 % BSA, bovine serum albumin, blocking solution (A7030; Sigma-Aldrich) for 15 min. Three washes of 1× PBS (5 min each) were carried out at every step. Cells were then incubated with mouse anti-α-tubulin at 1:500 (T6199; Sigma-Aldrich) overnight at 4 °C in the dark under humidity conditions. The alpha-tubulin was revealed with Alexa Fluor 568 nm anti-mouse antibody (A11004; Thermo Fisher Scientific) at 1:200, while the BIOT-NFL-peptide was revealed with Streptavidin Alexa Fluor 488 nm (S11223; Thermo Fisher Scientific) at 1:200 for 1 h each one. Subsequently, cells were counterstained with DAPI, 4′6-diaminido-2-phenylindole, solution (D9542; Sigma-Aldrich) at 1:300 for 20 min, and finally, coverslips were inverted and mounted with Prolong Gold Antifade reagent (P36930; Thermo Fisher Scientific). Experiments were repeated at least three times. Observations were carried out with a confocal microscope (Leica TCS SP8; Leica Biosystems, Nanterre, France).

### 2.5 Preparation and characterization of lipid nanocapsules loaded with Colchicine

#### 2.5.1 Lipid nanocapsules formulation

50-nm diameter LNCs were prepared (at a concentration of 200 mg/mL) according to the previously reported phase-inversion method developed by our laboratory in 2002 (Heurtault et al., 2002). All the mixture components (1.028 g Labrafac®, 0.075 g Lipoïd® S100, 0.846 g Kolliphor® HS15, 0.089 g NaCl, and 2.962 mL Milli-Q water) were mixed under magnetic stirring for approximately 5 min to create an oil-in-water emulsion. Three cycles of progressive heating (85 °C) and cooling (60 °C) were performed under magnetic stirring to change the emulsified system from o/w emulsion at low temperature into w/o emulsion at high temperature. During the last cooling cycle (around 60 °C), a volume of Milli-Q water (at 4 °C) was added into the emulsion to stabilize the particles and obtain lipid nanocapsules suspension. Then, slow magnetic stirring was applied to the formulation for 5 min until reaching room temperature. Non-loaded LNCs (Blank-LNCs) and drug-loaded LNCs were prepared using this method.

#### 2.5.2 Colchicine-loaded lipid nanocapsules (Col-LNCs)

Based on a solubility test of Colchicine in Labrafac oil, an accurately weighed amount of Colchicine was solubilized in the Labrafac® and sonicated for 15 min. Then, the Labrafac® was centrifuged (3000 rpm/ 10 min) and the oil supernatant containing soluble Colchicine was removed and used to formulate lipid nanocapsules loaded with Colchicine. The amount of soluble Colchicine in the Labrafac® was measured by UPLC method as previously described. Kolliphor® HS15, Lipoïd®, NaCl, and Milli-Q water were added to Labrafac containing Colchicine, and the formulation was performed as described above to obtain (Col-LNCs) in which the Colchicine is incorporated in the oily core (Labrafac).

Another type of lipid nanocapsules loaded with Colchicine was prepared by adding an equivalent amount of Col in the water used in the last cycle of formulation process (Water-Col-LNCs). Kolliphor® HS15, Lipoïd®, NaCl, Labrafac® and Milli-Q water were mixed and heated under magnetic stirring, and three cycles of progressive heating and cooling were carried out as described above. The deionized water containing Colchicine was added during the last cooling cycle (at 60–65 °C) to obtain Water-Col-LNCs.

#### 2.5.3 Characterization of LNC formulations

Nanocapsules were analyzed using a Malvern Zetasizer Nano Serie DTS 1060 (Malvern Instruments S.A., Worcestershire, UK). Mean particle size, polydispersity index (PDI), and zeta potential were measured for all different formulations (Blank-LNCs, Col-LNCs, and Water-Col-LNCs) coupled or not with BIOT-NFL-peptide. For the measurement, the formulations were diluted at 1:60 (*v*/v) in MilliQ water to ensure a convenient scattered intensity on the detector. Three consecutive measurements at 25 °C were performed.

To determine the Colchicine loading in the nanocapsules, total and encapsulated amount of Colchicine in the formulations were measured by UPLC method as described before. First, Colchicine-loaded lipid nanocapsules were purified by ultracentrifugation using an Ultracentrifuge (Beckman Coulter*,* USA) for 6 h at 20 °C and 55000 rpm to separate nanocapsules from aqueous solution. After 6 h, the lipidic fraction (lipid nanocapsules loaded with Colchicine) and the aqueous fraction (containing the Colchicine non encapsulated) were collected (Fig. S3). Then, the lipidic fraction was resuspended in water to yield dispersion nanocapsules. The resultant LNCs were then diluted 1:50 (v/v) in ethanol, to release the encapsulated Colchicine from the nanocapsules by breaking them, for the UPLC analysis. Aqueous fraction was also analyzed. The non-purified formulation (before ultracentrifugation) was also diluted 1:50 (v/v) in ethanol and analyzed by UPLC to determine the total amount of Colchicine in the formulation. The encapsulation efficiency was defined as:

Encapsulation efficiency (%) = Encapsulated Colchicine / Total Colchicine x 100

#### 2.5.4 Transmission Electron Microscopy (TEM)

Ultrastructural characterization of LNCs was investigated by transmission electron microscopy at the Microscopy Rennes Imaging Center platform (MRic TEM, Rennes, France). Four microliter samples were deposited to glow-discharged electron microscope grids for 1 min and were negatively stained with 2 % uranyl acetate for 10 s. The samples were observed using a 200 kV electron microscope (Tecnai G2 T20 Sphera, FEI) equipped with a 4 k × 4 k CMOS camera (model TemCam-F4160, TVIPS). Micrographs were acquired using the camera in binning mode 1.

#### 2.5.5 *In vitro* release study

The release kinetics of Colchicine from lipid nanocapsules loaded with Colchicine (Col-LNCs and Water-Col-LNCs) were evaluated in water. About 200 μL of each formulation was deposited into Pur-A-Lyzer® dialysis tube (10–250 μL) with molecular weight cut-off 6–8 kDa from Sigma-Aldrich, and then the tube was completely flooded in a beaker containing 10 mL water to reach the “sink” conditions. The experiment was performed at 37 °C under continuous stirring for 24 h. A control of free-Colchicine solution prepared at a concentration equivalent to the concentration of Colchicine loaded in the LNCs was also placed in a dialysis tube and dialyzed in the same manner. Several samples were removed from the outside of the dialysis membrane at defined time points (until 24 h) and the volume of each sample was replaced by an equal volume of water to maintain the sink conditions. Colchicine content was quantified in all samples by the UPLC method as described above. Concentration values were used to generate the Colchicine release profile. The experiment was performed in triplicate.

### 2.6 Coupling of Col-LNCs with BIOT-NFL-peptide

Lipid nanocapsules suspension (loaded or not with Colchicine) was incubated for 24 h with BIOT-NFL dissolved in Milli-Q water (at 1:1 ratio) under slow magnetic rotation at room temperature. The final concentration of BIOT-NFL and LNCs was 0.25 mM and 10 mg/mL respectively. A control of LNCs non-coupled with BIOT-NFL was also prepared by incubating LNCs with an equivalent volume of Milli-Q water in the same conditions. Later, LNCs with or without BIOT-NFL were characterized (size, polydispersity index and zeta potential) as previously described and visualized by electron microscope.

### 2.7 Cryo-Electron Microscopy (cryo-EM)

LNCs loaded with Colchicine and coupled with BIOT-NFL-peptide were observed by Cryo-electron microscopy to investigate the interaction between the nanocapsules and nanofibers formed by the peptide. The observations were performed at the Microscopy Rennes Imaging Center platform (MRic TEM, Rennes, France). The samples were deposited to glow-discharged electron microscope grids followed by blotting and vitrification by rapid freezing and were observed using a 200 kV electron microscope (Tecnai G2 T20 Sphera, FEI) equipped with a 4 k × 4 k CCD camera (model TemCam-F4160, TVIPS). Micrographs were acquired under low electron doses using the camera in binning mode 1 and at a nominal magnification of 25,000×.

### 2.8 *In vitro* cytotoxicity of Colchicine-loaded lipid nanocapsules coupled with BIOT-NFL on cancer cells

The cytotoxicity of peptide-coupled with Col-LNCs (BIOT-NFL/Col-LNCs) was evaluated on rat F98 glioblastoma cells by MTT assay as described previously (Griveau et al., 2022). Briefly, the initial suspension of peptide-lipid nanocapsules (0.25 mM BIOT-NFL/10 mg/mL Col-LNCs) was diluted in cell culture media into different concentrations with respect to the Colchicine loading in the nanocapsules. Five concentrations of Col-LNC were tested (equivalent to 0.01–0.015 - 0.02 - 0.03 and 0.15 μM of Colchicine) and corresponding to (0.1–0.2 - 0.3 - 0.5 and 2.5 μM) of the coupled peptide. Col-LNCs suspension (without the peptide) was also tested at the same concentrations equivalent to Colchicine. Cells treated with increasing concentrations of free Colchicine, free BIOT-NFL and Blank-LNCs coupled with BIOT-NFL at equivalent concentrations were used as controls. Three parallel wells were performed for each condition. After 72 h of treatment, 10 μL of MTT working solution was added to each well and an MTT assay was realized as described above. Three independent experiments were conducted. The same experiment was done with the two different cell lines (MIA PaCa-2 and SH-SY5Y) in triplicate in order to compare to F98 glioblastoma cells.

### 2.9 Analysis of the uptake of BIOT-NFL-peptide and the effect of BIOT-NFL/Col-LNCs by confocal microscopy

Immunofluorescence analysis of F98 cells treated with lipid nanocapsules loaded with Colchicine and coupled or not with BIOT-NFL was also performed (as described previously). Rat F98 glioblastoma cells were treated with BIOT-NFL coupled with Col-LNCs and compared to cells treated with BIOT-NFL/Blank-LNCs and free BIOT-NFL at equivalent concentrations. All conditions were observed by confocal microscopy. Only confocal images of cells treated with BIOT-NFL/Col-LNCs at 0.02 μM Colchicine concentration will be presented here. The cellular uptake of peptide was also investigated by microscopic examinations.

### 2.10 Statistical analysis

Statistical analyses were performed using GraphPad Prism software. Results were expressed as mean values ± SEM. All experiments were repeated at least three times. A Student's *t*-test was used for statistical analysis. Both asterisks and hash symbols were used to denote significance levels in different experimental conditions or datasets, as detailed in the figure legends (*p < 0.05; **p < 0.005 and ***p < 0.001) and (#p < 0.05; ##p < 0.005; ###p < 0.001).

## 3 Results

### 3.1 The effect of BIOT-NFL-peptide combined with free Colchicine on glioblastoma cell viability

**Fig. 1:**
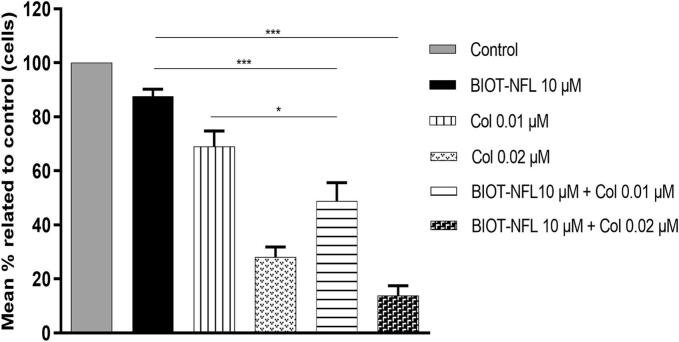
MTT assay results of F98 glioblastoma cells treated with BIOT-NFL-peptide and free Colchicine for 72 h. Rat F98 glioblastoma cells were treated with the peptide (10 μM BIOT-NFL), two different concentrations of Colchicine (0.01 μM Col) and (0.02 μM Col), and the peptide combined with Colchicine (10 μM BIOT-NFL + 0.01 μM Col) and (10 μM BIOT-NFL + 0.02 μM Col). A MTT assay was performed, after 72 h of treatment at 37 °C, to evaluate the *in vitro* toxic effect of BIOT-NFL and Colchicine on GBM cells, and a control group (cells without treatment) was done for each experiment. The experiment was repeated at least three times independently (*n* = 3). (*p < 0.05; **p < 0.005 and ***p < 0.001).

MTT assay was used to measure cellular metabolic activity as an indicator of cell viability. The *in vitro* toxic effect of these two agents, BIOT-NFL-peptide and Colchicine, was investigated on glioma cells as described above. F98 rat glioblastoma cells were first treated with increasing concentrations of Colchicine. Cellular mitochondrial activity decreased in a concentration-dependent manner after colchicine treatment for 72 h (data not shown), allowing determination of EC50. Based on the EC50 of Colchicine on F98 cells, two different Colchicine concentrations were selected for this study (0.01 and 0.02 μM). The BIOT-NFL was tested at a concentration of 10 μM corresponding to ∼80 % of F98 cell viability. F98 cells were treated with each compound alone (10 μM BIOT-NFL, 0.01 μM Col, and 0.02 μM Col) or with BIOT-NFL combined with Colchicine at the two different concentrations (10 μM BIOT-NFL with 0.01 or 0.02 μM of Col). Interestingly, increased inhibition of cell growth was revealed by the MTT assay when cells were treated with BIOT-NFL peptide and Colchicine, indicating that the effect of these agents is better when combined (Fig. 1).

#### 3.1.1 Microtubule network disruption

**Fig. 2:**
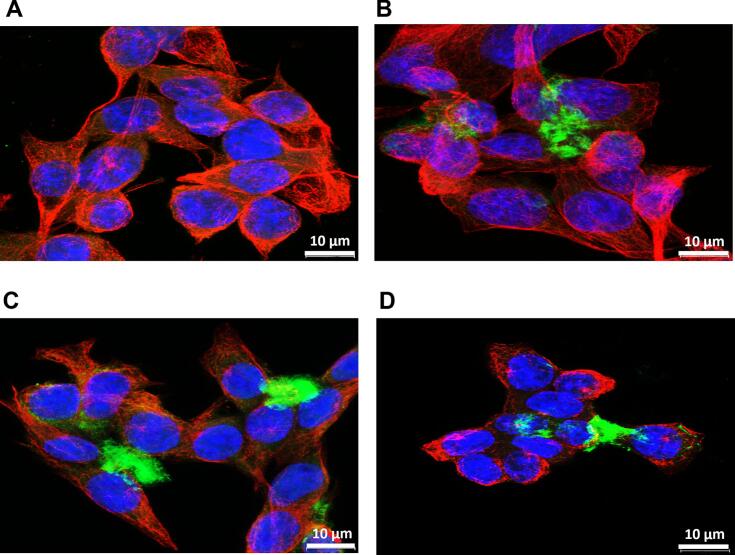
Immunofluorescence staining of microtubule network in rat glioblastoma cells incubated with BIOT-NFL-peptide and free Colchicine. Confocal microscopy of rat F98 glioblastoma cells treated with BIOT-NFL-peptide with or without Colchicine for 6 h. (A): A control of non-treated cells. (B): Cells treated with 10 μM of BIOT-NFL. (C), (D): Cells treated with 10 μM of BIOT-NFL combined with Colchicine at 0.01 μM and 0.2 μM, respectively. F98 glioblastoma cells immunostained with anti-α-tubulin (red) to reveal the microtubule network. Nuclei were labelled with DAPI (blue). The BIOT-NFL-peptide was visualized in green using Streptavidin Alexa Fluor. Scale bars: 10 μm. The experiment was repeated at least three times independently (n = 3). (For interpretation of the references to colour in this figure legend, the reader is referred to the web version of this article.)

To further evaluate the effect of the BIOT-NFL-peptide combined with Colchicine on F98 glioblastoma cells, their microtubules network was investigated by tubulin immunofluorescence analysis. Cells were treated with 10 μM of BIOT-NFL alone or combined with Colchicine at the two different concentrations (0.01 and 0.02 μM). A control of cells without treatment has always been done. In untreated cells, a well-organized microtubules network with red fluorescence was observed (Fig. 2A). No major difference was noticed between non-treated cells and cells treated with 10 μM of BIOT-NFL (Fig. 2B). However, a clear alteration of the microtubule network and much less cells were observed when they were treated with the combination of Colchicine and the BIOT-NFL-peptide (Fig. 2C and D). These results revealed that BIOT-NFL treatment combined with Colchicine promoted the destruction of microtubules, which in turn prevented cells from dividing, causing their death. Thus, confocal microscope observations confirmed the results obtained by MTT assay.

The uptake of BIOT-NFL-peptide was also investigated by confocal microscopy. Interestingly, the BIOT-NFL-peptide (visualized in green) was localized mainly on F98 cells, and an amount of peptide was detected inside cells as demonstrated in the 3D image of confocal microscopy (Fig. S2). These results further confirmed the ability of this peptide to target GBM cells and stick on their surface.

#### 3.1.2 Development and characterization of Colchicine-loaded lipid nanocapsules Colchicine-loaded lipid nanocapsules formulations

Before performing the formulation, a solubility test of Colchicine in different oils was first done, and two oils were previously chosen to solubilize the Colchicine: Labrasol and Labrafac. Firstly, a formulation of lipid nanocapsules loaded with Colchicine was performed using these two oils (80 % Labrafac and 20 % Labrasol). However, this formulation was not stable, and the presence of Labrasol oil in the formulation does not allow the interaction between lipid nanocapsules and BIOT-NFL when they are coupled together (data not shown), thus, this formulation was discarded.

LNCs formulation was then performed after solubilization of Colchicine only in the Labrafac oil. Another formulation approach was also tested by adding the Colchicine at the last cycle of formulation process. Hence, in this work, two different formulations of Colchicine were developed: (Col-LNCs) in which the Colchicine was solubilized in the Labrafac oil and (Water-Col-LNCs) in which the Colchicine was added in the water during formulation. Blank-LNCs, free-drug-lipid nanocapsules, were also prepared (B-LNCs).

**Fig. 3:**
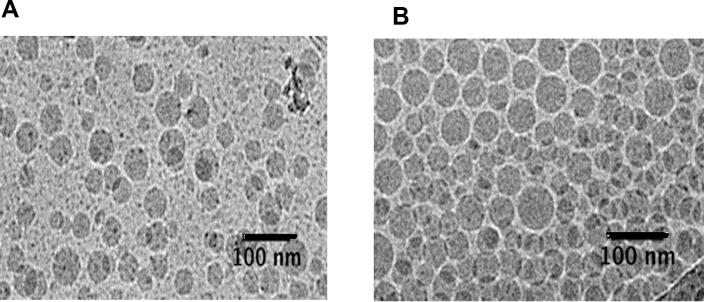
Morphological analysis of Colchicine-loaded lipid nanocapsules. Cryo-electron microscopy images of lipid nanocapsules loaded with Colchicine. (A): Col-LNCs (Colchicine solubilized in the oily core). (B): Water-Col-LNCs (Colchicine added in the aqueous phase). Samples were deposited to glow-discharged electron microscope grids followed by blotting and vitrification by rapid freezing and were observed using Cryo-EM. Scale bar: 100 nm.

Lipid nanocapsules of Colchicine with a narrow particle size between 50 and 55 nm were successfully obtained following the two different formulations. No precipitation of Colchicine was observed during LNCs preparation in the two cases. Table 1 shows the characteristics of different formulations. The polydispersity index (PI) of all formulations was <0.1 which demonstrates the mono dispersity of the preparations. Zeta potentials were negative for all LNCs formulations. The size of particles was confirmed by transmission electron microscope analysis, and a spherical morphology of LNCs (Col-LNCs and Water-Col-LNCs) was observed as showing in Figs. 3A and B respectively.

**Table 1:** Characterization (size, polydispersity index and zeta potential) of Blank-lipid nanocapsules and Colchicine-loaded lipid nanocapsules coupled or not with BIOT-NFL-peptide.

Formulations	Size (nm)	PDI	Potential (mV)	Encapsulation efficiency (%)
Blank-LNCs	53.2 ± 0.3	0.042 ± 0.007	−4.01 ± 0.9	–
Col-LNCs	56.6 ± 0.81	0.053 ± 0.011	−3.55 ± 1.3	29.7 ± 2.7 %
BIOT-NFL/Col-LNCs	57.3 ± 0.75	0.070 ± 0.010	−0.77 ± 1.01	–
Water-Col-LNCs	58.1 ± 1.01	0.050 ± 0.009	−5.18 ± 0.72	31.4 ± 1.53 %
BIOT-NFL/Water-Col-LNCs	58.7 ± 0.63	0.062 ± 0.004	−0.31 ± 0.50	–

Physicochemical characteristics and encapsulation efficiency of Col-LNCs and Water-Col-LNCs alone or coupled with BIOT-NFL. An incubation between the peptide and lipid nanocapsules loaded with Colchicine (Col-LNCs or Water-Col-LNCs) was realized overnight at room temperature under gentle magnetic stirring. Three characteristics were measured: the size in nanometer (nm), the PI (polydispersity index) and the zeta potential in millivolt (mV). Experiments were performed at least in triplicate.

#### 3.1.3 Entrapment of Colchicine in lipid nanocapsules

To ensure the loading of Colchicine in the nanocapsules, the two formulations were purified (by ultracentrifugation as described above) to separate encapsulated and non-encapsulated Colchicine. Blank-LNCs were also centrifuged, and their fractions were used as controls. After purification, the lipidic (LNCs) and aqueous fractions of the formulation were collected (Fig. S3) and analyzed by UPLC to measure the amount of encapsulated and non-encapsulated Colchicine, respectively. The purified formulation was diluted in ethanol to release the encapsulated Colchicine from the nanocapsules before analysis. An amount of Colchicine was detected and quantified by UPLC in the lipidic phase of the purified formulation, indicating that the Colchicine was successfully loaded in the nanocapsules. Colchicine not encapsulated in the formulation was found in the aqueous fraction after ultracentrifugation. The total amount of Colchicine in the non-purified formulation (before ultracentrifugation) was also measured. All Colchicine measurements were performed by the UPLC-UV method as described above. Then, the encapsulation efficiency (EE %) was determined as described before.

An encapsulation efficiency of Colchicine about 30 % was obtained for the different formulations (Table 1). It appears that the two formulations (Col-LNCs and Water-Col-LNCs) were successfully loaded with Colchicine. The purified formulation was charged with 300 μM of Colchicine loaded into nanocapsules. The coupling with BIOT-NFL does not change the size or PI of lipid nanocapsules.

#### 3.1.4 *In vitro* release study

The release studies are often performed to predict how a delivery system might work in the organism after its administration. *In vitro* release study of Colchicine was carried out by a dialysis method. The two formulations were dialyzed in plain water and compared to free Colchicine solution at equivalent concentration. Due to the tendency of the NFL-peptide to aggregate in PBS, all previous experiments were conducted in aqueous solution (Alnemeh-Al Ali et al., 2022). Accordingly, PBS was avoided in the present experiment, in line with established protocols. However, the experiment was carried out at 37 °C under continuous stirring for 24 h to closely mimic biological conditions. The water used was ultrapure Milli-Q, specifically intended for analytical techniques in particular UPLC, with a pH typically close to neutral (approximately 7).

**Fig. 4:**
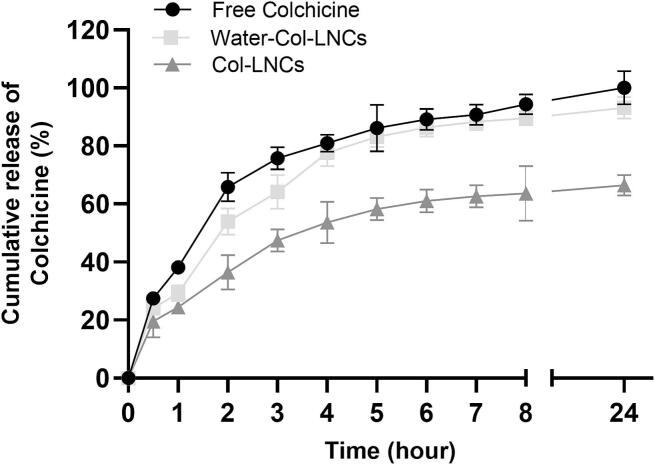
Release profile of Colchicine from lipid nanocapsules loaded with Colchicine. *In vitro* release study of Colchicine from lipid nanocapsules formulations (Col-LNCs: Colchicine solubilized in the Labrafac oily core, and Water-Col-LNCs: colchicine added in the aqueous phase). The cumulative release of Colchicine from lipid nanocapsules was compared to that of free-Colchicine at equivalent concentration. 200 μL of each formulation was deposited into dialysis tube (6–8 kD MWCO Pur-A-Lyzer) and then placed in a beaker containing 10 mL of Milli-Q water under continues stirring at 37 °C. Samples were taken from the outside of dialysis tube at different time points (until 24 h). The figure represents the quantification of Colchicine outside the dialysis membrane by UPLC measurements. Data are presented as mean ± SEM. The experiment was repeated at least three times independently (n = 3).

A slower release from Col-LNCs than free Colchicine or Water-Col-LNCs was revealed as seen in Fig. 4. Approximately 70 % of the Colchicine was released from Col-LNCs after 24 h, whereas a total release of Colchicine was detected from the two other conditions (free Colchicine and Water-Col-LNCs). The Colchicine release was longer from Col-LNCs than Water-Col-LNCs, indicating that the retention of Colchicine in LNCs seems to be better when it was solubilized in the Labrafac oil. The coupling of BIOT-NFL-peptide with lipid nanocapsules did not change the release rate of Colchicine (data not shown). Based on these results, Col-LNCs (in which the Colchicine was added in the oily core of the formulation) was selected for the next section of this work.

### 3.2 Colchicine-loaded lipid nanocapsules coupled with BIOT-NFL-peptide coupling between BIOT-NFL and Col-LNCs

The ideal nanotherapy should have specific targeting to tumour cells, to minimize or avoid off-target effects of the active therapeutic agent on healthy tissues. The selectivity of BIOT-NFL against GBM cells as well as its ability to form peptide-nanofibers, previously demonstrated in our prior work (Alnemeh-Al Ali et al., 2022; Griveau et al., 2022), motivated our investigation into a potential new nanosystem that combines BIOT-NFL with lipid nanocapsules loaded with Colchicine (Col-LNCs). Thus, the interaction between peptide-nanofibers and Col-LNCs was investigated here. In this purpose, several concentrations of Col-LNCs and BIOT-NFL were coupled together and the suspension of peptide-nanofibers/ nanocapsules was then examined by Cryo-electron microscopy.

**Fig. 5:**
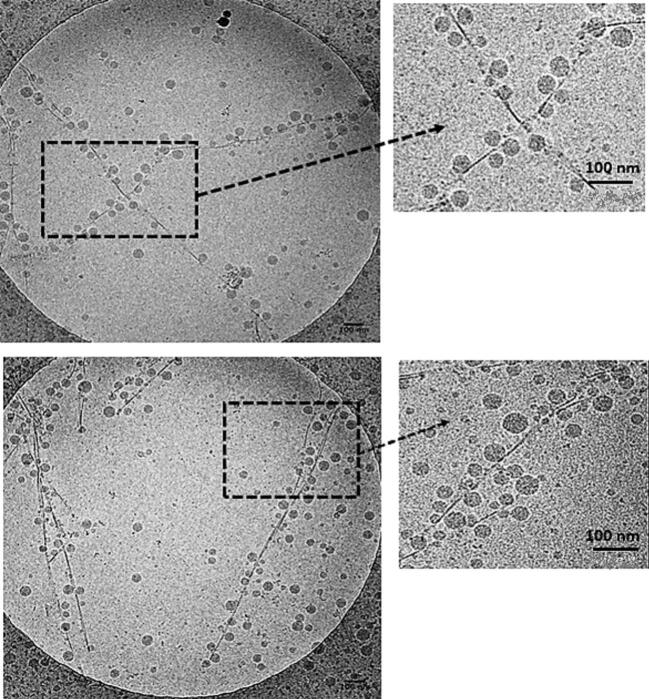
Cryo-electron microscopy images of BIOT-NFL-peptide nanofibers decorated with Colchicine-loaded lipid nanocapsules. Observations by Cryo-electron microscopy of Colchicine-loaded lipid nanocapsules coupled with BIOT-NFL reveal the nanofibers formed from BIOT-NFL peptide at this concentration and the adsorption of spherical Col-LNCs all long peptide-nanofiber. The coupling between Col-LNCs suspension (10 mg/mL) and BIOT-NFL peptide (0.25 mM) was realized at 1:1 ratio by gentle magnetic stirring for 24 h. Samples were deposited to glow-discharged electron microscope grids followed by blotting and vitrification by rapid freezing and were observed using Cryo-EM. Scale bar: 100 nm.

The optimal ratio of coupling between peptide-nanofibers and Col-LNCs was defined previously by Cryo-EM observations at (0.25 mM BIOT-NFL/10 mg/mL Col-LNCs) (Griveau et al., 2022). Interestingly, BIOT-NFL-nanofibers decorated with lipid nanocapsules loaded with Colchicine were observed by cryo-electron microscopic examination. As demonstrated in Fig. 5, the majority of Col-LNCs were adsorbed along the nanofibers formed by the BIOT-NFL peptide; however, few free LNCs have always been found. Another concentration of Col-LNCs was coupled with the peptide at the same ratio (0.25 mM BIOT-NFL-peptide/5 mg/mL Col-LNCs) and showed the same interaction (data not shown). Hence, Cryo-EM micrographs indicated a possible new delivery nanosystem (nanofibers / nanocapsules) that might be used to target GBM.

#### 3.2.1 Cytotoxicity of BIOT-NFL-coupled Col-LNCs on cancer cells

**Fig. 6:**
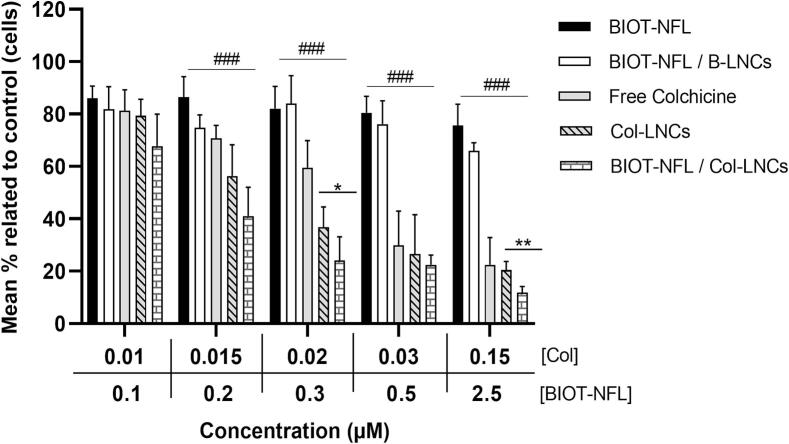
*In vitro* cytotoxicity study of Colchicine lipid nanocapsules coupled or not with BIOT-NFL on rat F98 cells. Effect of various formulations, Col-LNCs (Col-loaded LNCs without the peptide), BIOT-NFL/Col-LNCs (Col-loaded LNCs coupled with BIOT-NFL) and BIOT-NFL/B-LNCs (blank-LNCs coupled with BIOT-NFL) on rat F98 glioblastoma cells after 72 h incubation. Cells were treated with free-Col and free-BIOT-NFL at equivalent concentrations. The response of cells was measured by MTT assay. Values are expressed as mean ± SEM. The experiment was repeated at least three times independently (n = 3). BIOT-NFL/Col-LNCs as compared with Col-LNCs (*) and as compared with BIOT-NFL/B-LNCs (_#_). (*p < 0.05; **p < 0.005 and ***p < 0.001), (^#^p < 0.05; ^##^p < 0.005 and ^###^p < 0.001).

The effect of lipid nanocapsules loaded with Colchicine (Col-LNCs) and coupled with BIOT-NFL was first evaluated on F98 rat glioblastoma cells by MTT assay (Fig. 6). The peptide-lipid nanocapsules (0.25 mM BIOT-NFL/10 mg/mL Col-LNCs), corresponding to 15 μM Colchicine concentration, was diluted with respect to the Colchicine loading in LNCs into different concentrations ranging from 0.01 to 0.15 μM of Colchicine. BIOT-NFL concentrations after dilution were ranging from 0.1 to 2.5 μM. Col-LNCs non-coupled with BIOT-NFL were also diluted in the same manner and applied on cells. The results obtained were compared with cells treated with BIOT-NFL alone, BIOT-NFL/B-LNCs and free Colchicine at equivalent concentrations.

The MTT assay showed that free BIOT-NFL as well as Blank-LNCs coupled with BIOT-NFL (BIOT-NFL/B-LNCs) did not display radical cytotoxicity on cells at these concentrations, indicating that the Blank-LNCs was biocompatible, and that BIOT-NFL peptide linked or not on the particle surface did not cause obvious toxicity at these low concentrations when it was administrated alone. On the opposite, Col-LNCs showed cytotoxic effects on F98 cells at all concentrations studied, with an increase in cytotoxicity as concentration increased. When Col-LNCs were coupled with the peptide (BIOT-NFL/Col-LNCs), their cytotoxicity was higher than Col-LNCs alone (without peptide), and greatly much higher when compared with non-loaded LNCs (B-LNCs) coupled with BIOT-NFL at same concentrations. Significant difference between Col-LNCs and BIOT-NFL/Col-LNCs was observed at 0.15 μM of Colchicine coupled with the highest concentration of peptide (2.5 μM). These results could be attributed to the BIOT-NFL-peptide, which facilitated Col-LNCs uptake by GBM cells, and thus increased the intracellular drug concentration.

#### 3.2.2 Confocal microscopy analysis

**Fig. 7:**
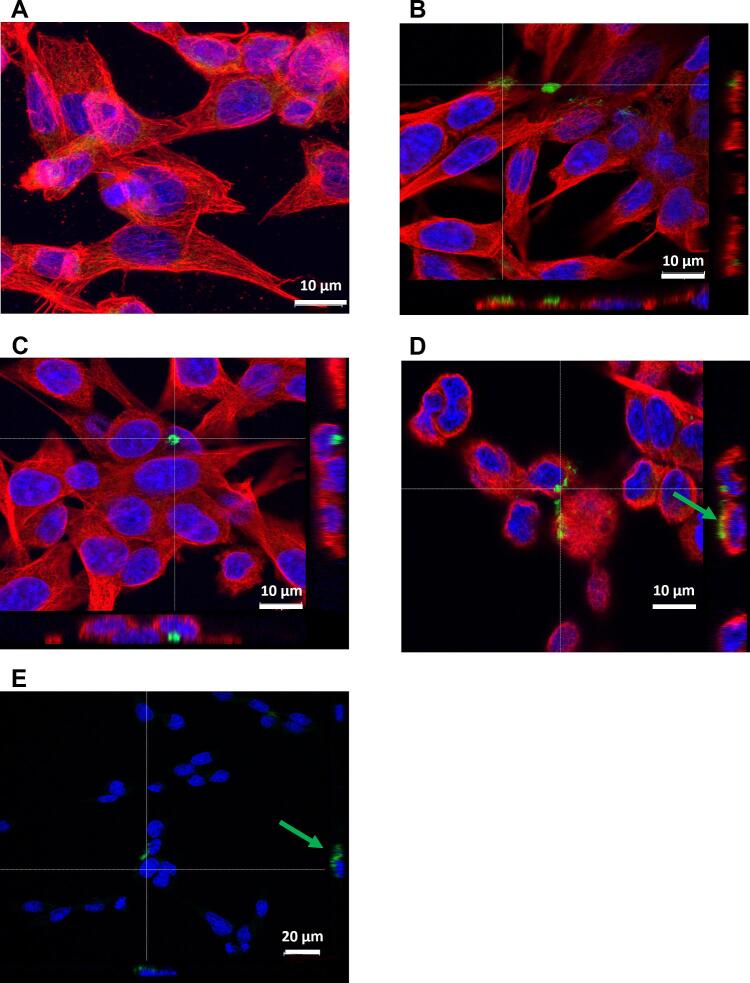
Confocal experiment to verify the cytotoxic effect of BIOT-NFL/Col-LNCs and the uptake of peptide in GBM. Confocal microscopy micrographs of F98 glioblastoma cells, (A): without treatment (control), or treated with; (B): BIOT-NFL, (C): BIOT-NFL/B-LNCs, (D) and (E): BIOT-NFL/Col-LNCs at 0.02 μM Colchicine concentration. The BIOT-NFL concentration was adjusted to be the same with or without nanoparticles. Cells were incubated 6 h at 37 °C with each treatment. BIOT-NFL was visualized in green using Streptavidin Alexa Fluor. Microtubules network was revealed in red using anti-α-tubulin, and nuclei were labelled in blue with DAPI. Scale bar: 10 μm and 20 μm. The experiment was repeated at least three times independently (n = 3). (For interpretation of the references to colour in this figure legend, the reader is referred to the web version of this article.)

The cytotoxicity of BIOT-NFL/Col-LNCs on GBM cells as well as the uptake of peptide was also examined by tubulin immunofluorescence analysis. Only images of cells treated with BIOT-NFL coupled-Col-LNCs (at 0.02 μM Colchicine concentration) were presented here. Cells were also treated with BIOT-NFL-peptide and BIOT-NFL coupled with Blank-LNCs as controls. BIOT-NFL-peptide linked or not with Blank-LNCs (Fig. 7B and C respectively) did not cause detectable alteration of microtubules network at this concentration compared to non-treated cells (Fig. 7A). When cells were treated with BIOT-NFL coupled-Col-LNCs, a spherical cell shape with a clear destruction of microtubules network was observed (Fig. 7D). For BIOT-NFL and BIOT-NFL/B-LNCs, the peptide concentration was adjusted to be the same (0.3 μM BIOT-NFL) as its concentration in the coupling (BIOT-NFL/Col-LNCs).

Interestingly, in all cases, BIOT-NFL (visualized in green) was attached on GBM cells, and an amount of peptide was every so often detected near the nucleus even at this low concentration of peptide, as seen in Fig. 7E. Here, only confocal images showing the peptide were presented, as at this low concentration of BIOT-NFL (0.3 μM), the peptide was not found in all scenes. Confocal micrographs indicate that BIOT-NFL binds strongly GBM cells and its coupling with Col-LNCs may probably target their delivery into these cells.

## 4 Discussion

Drug delivery has always been considered as a major contest in the treatment of glioblastoma. Although several researchers have made progress in finding effective treatments for this cancer, the challenges of targeting only tumour cells and ensuring therapeutic concentrations at the target site are still highlighted. One of the strategies used to address current challenges in GBM treatment and increase the benefits of therapy is the application of nano-approaches for the delivery or co-delivery of anti-cancer drugs (Mujokoro et al., 2016; Ganipineni et al., 2018; Alphandéry, 2020). Nanostructures have recently become the main attraction materials for applications in drug delivery. Thanks to their physicochemical properties and nano scale sizes, these structures can interact in unique fashion with biological systems and penetrate several biological barriers in the organism (Moghimi et al., 2005; Peer et al., 2007). Two decades have passed since the first nanoparticles (NP)-based cancer treatment was approved by the FDA, with an increasing number of preclinical and clinical trials now ongoing, including many for GBM (Zhao et al., 2020). However, most of nanomedicine-based glioma therapy remain perfectible before employment in the clinics. Therefore, we discuss here a new nano-based delivery system including GBM-targeting peptide and lipid nanocapsules for the treatment of this cancer.

A potential new nanosystem arranged of BIOT-NFL-nanofibers coupled with lipid nanocapsules loaded with anti-microtubule agent (Colchicine) was developed in this work to target GBM. Colchicine (Col) is a cytotoxic drug that binds the tubulin at Colchicine Binding Site (CBS) and prevents the polymerization of microtubules inducing cell death (McLoughlin and O'Boyle, 2020). The most favourable factor of Colchicine and other Colchicine Binding Site Inhibitors (CBSIs) comparing to other anti-microtubule agents, is that most of these drugs have no multidrug resistance (MDR) issues (Yang et al., 2018). Several CBSIs have been identified as potential anticancer agents for clinical studies due to their ability to overcome Pgp/β-III tubulin mediated drug resistance as well as their anti-angiogenic or anti-vascular actions (Lu et al., 2012; Arnst et al., 2019).

BIOT-NFL-peptide is a promising GBM-targeting peptide that previously demonstrated its selective and massive entry in glioblastoma cells by endocytosis (Lépinoux-Chambaud and Eyer, 2013). NFL-peptide can also exhibit an anti-microtubule activity on GBM cells at appropriate concentrations (Berges et al., 2012a). Additionally, the presence of NFL-peptide on the surface of lipid nanocapsules has shown to increase their cellular uptake and their efficiency in an *in vitro* as well as *in vivo* glioma models. The prior findings further suggest the ability of NFL-peptide to penetrate the BBB following intravenous administration (Balzeau et al., 2013). A recent study conducted by our team as part of another research project also demonstrated that the NFL-peptide promotes the passage of functionalized particles across an *in vitro BBB* model. An *in-vivo* approach is currently underway and appears to confirm the data obtained *in-vitro* (data not shown), but the molecular mechanism allowing this transport is not yet fully understood (Mellinger et al., 2023).

All these properties recommend this peptide as an innovative and promising therapeutic candidate for drug delivery and cancer treatment. In this work, our study revealed an enhancement of anti-tumour activity when BIOT-NFL was combined with Colchicine and administered into rat F98 glioblastoma cells. As demonstrated in Fig. 1, Fig. 2, a stronger inhibition of GBM cells with a clear destruction of their microtubules network were observed in cells treated with 10 μM BIOT-NFL combined with Colchicine at two different concentrations (0.01 and 0.02 μM). When microtubules are disrupted, the formation of mitotic spindles is hampered, and cells enter apoptosis (Field et al., 2014). Moreover, confocal examinations showed that BIOT-NFL (visualized in green) was mainly stuck on GBM cells with the presence of a quantity of peptide detected inside the cells (Fig. S2), which confirm the GBM targeting ability of BIOT-NFL.

Based on these observations, we intended here to develop lipid nanocapsules loaded with Colchicine to facilitate the administration of this compound in the organism as well as its coupling with BIOT-NFL-peptide to target its delivery to cancer cells. An example of targeted nano-delivery system of Colchicine, using nanoparticles loaded with Col and coated with folic acid chitosan-glycine complex as a targeting ligand for cancers, has recently been reported in the literature. The resulting nanoformulation was found to be effective in several cell lines with targeted delivery (AbouAitah et al., 2020).

Lipid nanocapsules (LNCs) are drug-delivery nanocarriers that have already demonstrated their ability to optimize the biodistribution and therapeutic effects of anti-cancer drugs, and improve cancer treatment (Pautu et al., 2021). LNCs were synthesized using a pharmaceutically acceptable excipient by a phase inversion process previously developed in our laboratory (Heurtault et al., 2002). Two different formulations of lipid nanocapsules loaded with Colchicine were developed in this work (Col-LNCs: in which the Colchicine was solubilized in the Labrafac oily core, and Water-Col-LNCs: in which the Colchicine was added with water at the last cycle of formulation process). DLS and TEM assessment were performed to characterize the particles and to determine their size (Table 1 and Fig. 3). LNCs loaded with Colchicine spherical in shape, with a size around 55 nm and negative surface charge were successfully obtained. No significant changes in mean particle size, polydispersity index, and zeta potential were observed during storage (data not shown).

The entrapment of Colchicine into LNCs was determined by UPLC measurements following the separation of encapsulated and non-encapsulated Colchicine in the formulation (by ultracentrifugation method) (Briot et al., 2017). No major difference was observed of the Colchicine loading in these two formulations; however, the *in vitro* release study of the formulations showed that the release of Colchicine was slower from Col-LNCs than the other formulation (Water-Col-LNCs) (Fig. 4). Our hypothesis was that the Colchicine might be localized in the shell of nanocapsules when it was added with water at the last cycle of the formulation (in Water-Col-LNCs formulation), and consequently its released should be faster (like the free Colchicine). Moreover, Water-Col-LNCs were physically stable for 3 months at 2–8 °C, while Col-LNCs were stable for at least 6 months when they were stored at the same conditions. These results suggested that the Colchicine was better encapsulated in the Col-LNCs, when the Colchicine was solubilized in the Labrafac and added at the beginning of the formulation process. These results might give some indication of the *in vivo* performance of the formulations, and thus this formulation (Col-LNCs) was selected for the following works. The attachment of BIOT-NFL did not change the release rate of Colchicine from nanocapsules (data not shown), and this can be easily explained as the coupling between peptide/LNCs occurs by adsorption on the surface of particle without interfering with the particle matrix (Balzeau et al., 2013). Due to their characteristics (size, shape, and surface features), LNCs are known to accumulate in the area around the tumour by passive targeting through the EPR (enhanced permeability and retention) effect (Morille et al., 2010). However, the passive tumour targeting does not promote the uptake of nanoparticles by tumour cells, consequently, the drug molecules carried by nanoparticles are released in the extracellular matrix and diffuse throughout the tumour region without necessarily targeting tumour cells. Therefore, directed delivery of nanoparticles into tumour cells is required to improve the effectiveness of treatment (Torchilin, 2010). For this reason, here we aimed to couple Colchicine-loaded lipid nanocapsules with BIOT-NFL-peptide that can target and penetrate GBM cells as previously demonstrated. The design of this nanosystem can aid the delivery of Col-LNCs to the tumour home by the help of the peptide.

The use of tumour-targeting peptides to target the delivery of nanoparticles for cancer therapy have been widely described in the literatures (Kebebe et al., 2018; Zhao et al., 2018). Many peptides such as RGD, Angiopep-2 and Tet-1 have been well used to target the delivery of nanoparticles for different applications and have shown great potential in preclinical studies (Friedman et al., 2013). In cancer therapy, specifically addressing tumour cells offer the ability to selectively destroy malignant cells, while sparing healthy tissue. BIOT-NFL-peptide, reported to have cell-penetrating properties, is promising for drug-targeting and cancer-therapy applications, since it can target GBM cells without major toxicity on healthy cells. A previous study by Paillard et al. (2010), reported that internalization of uncoupled LNCs was not preferentially targeted to GBM cells and also entered healthy astrocytes, whereas the coupling of LNCs with NFL-peptide has preferentially targeted them to GBM cells and enhanced their cellular internalization compared to healthy cells. Moreover, the internalization of NFL-coupled LNCs in GBM cells was mediated by an endocytosis pathway like that of the NFL-peptide, indicating that LNCs entry is probably facilitated by the help of peptide (Paillard et al., 2010; Karim et al., 2018). The ability of NFL-peptide to interact with LNCs and enhance their internalization in GBM cells has also been explored and proven in our previous work (Griveau et al., 2022). All these findings were crucial and supported the use of NFL-peptide as a ligand for GBM-targeting nanoparticles.

Accordingly, the formation of BIOT-NFL-nanofibers that demonstrated in our prior work, can be precisely programmed for the design of smart drug delivery system for the effective targeting of GBM (Alnemeh-Al Ali et al., 2022). The application of nanofibers for the delivery of therapeutics to tumour site have been widely reported in the literature (Abid et al., 2019). For example, a study by Zhang et al. (2013), has explored the use of TAT-conjugate-nanofibers for the delivery of paclitaxel into cancer cells. Hence, BIOT-NFL-peptide was sought to be used as an active targeting agent for the delivery of GBM therapeutics.

Interestingly, a new potential nanosystem composed of BIOT-NFL-nanofibers decorated with lipid nanocapsules loaded with Colchicine was developed here. As shown in Fig. 5, an interaction between Col-LNCs and BIOT-NFL-nanofibers was remarkably observed by Cryo-electron microscopy examination. The ratio between peptide/LNCs may influence this interaction (Zhu et al., 2005). Thus, several concentrations of BIOT-NFL and Col-LNCs were studied, and the optimal interaction was observed with 10 mg/mL of LNCs coupled with 0.25 mM of BIOT-NFL. The presence of Col-LNCs alongside BIOT-NFL-nanofibers can facilitate the targeted uptake of LNCs by glioblastoma cells owing to the selectivity of BIOT-NFL against these cells. This attachment between peptide/nanocapsules is thought to be due to electrostatic interactions between the positively charged peptide at physiological pH (pI = 10.3) and the negative zeta potential (−3.5 mV) at the surface of lipid nanocapsules. As demonstrated in Table 1, the coupling of lipid nanocapsules with BIOT-NFL (by an incubation for 24 h) has decreased the zeta potential of LNCs coupled with peptide (approximately −3 mV), indicating that the adsorption between peptide/lipid nanocapsules results from electrostatic interactions. As this attachment is caused by adsorption, the structure of BIOT-NFL may still be available for other preferential functions of the peptide (Berges et al., 2012b). Similar observations have also been demonstrated in another study (Balzeau et al., 2013), which indicated that the NFL interacts with the polar PEG chains of the Kolliphor, whereas Carradori et al. (2016) suggested that the interaction was possibly due to a combination of electrostatic forces and other weak forces *i.e.* Van der Waal's forces and hydrophobic forces.

To further evaluate whether the NFL-peptide is rapidly removed from the LNC surface after dilution, other studies have also performed dialysis tests, and the free peptide concentration was quantified from the receiver compartment by HPLC. Dialysis experiments were performed in distilled water and not in PBS because PBS causes the aggregation of the NFL-Biot peptide (data not shown). The desorption of NFL from the LNC surface was slow and gradual, with only 33.6 % of the peptide released after 67 h. These results indicated that the peptide adsorption percentage is very high and strongly attached to the LNC surface (Karim et al., 2018). Further research is underway to better understand the underlying molecular mechanisms.

After optimizing the ratio that permit the best interaction between BIOT-NFL nanofibers and Col-LNCs, BIOT-NFL/Col-LNCs were incubated with F98 cells, with respect to the Colchicine concentration loaded into the nanocapsules, to evaluate their anti-tumour effect. The *in vitro* cytotoxicity study indicated that BIOT-NFL coupled with Col-LNCs exhibited higher activity of inhibiting GBM cells than non-coupled Col-LNCs (Fig. 6). This inhibition was more significant with the highest concentration of peptide (2.5 μM BIOT-NFL coupled with 0.15 μM Colchicine). These results could be attributed to the facilitated uptake of BIOT-NFL/Col-LNCs by GBM cells mediated by the peptide. However, the difference between coupled- and non-coupled-Col-LNCs was not significant in all studied concentrations, probably explained by the low concentrations of BIOT-NFL after dilution (Yu et al., 2010). Hence, additional experiments with different LNCs/peptide concentrations will be proceeding in order to optimize the peptide concentration.

To further confirm the targeting effect of BIOT-NFL on GBM cells, the same experiment was performed on two other different cell lines (MIA PaCa-2) and (SH-SY5Y). The effect of various formulations, BIOT-NFL/B-LNCs, Col-LNCs and BIOT-NFL/Col-LNCs, as well as free Colchicine, and BIOT-NFL at equivalent concentrations was shown on MIA PaCa-2 and SH-SY5Y cells in Figs. S4 and S5, respectively. Even though MTT assay results showed cytotoxic effects of Col-LNCs on these cells, no significant difference in cytotoxicity levels between cells treated with Col-LNCs and those treated with BIOT-NFL/Col-LNCs was showed at any concentration. These results suggest that the coupling of BIOT-NFL with Col-LNCs does not seem to improve the internalization of nanocapsules in these cells, which may argue the targeting of BIOT-NFL to GBM cells, and not on these cells (MIA PaCa-2 and SH-SY5Y). Nevertheless, an additional internalization test on the two cell lines will be necessary to confirm these results.

In Fig. 7, confocal images clearly showed the uptake of BIOT-NFL by GBM cells with the presence of an amount of peptide inside the cell, confirming the targeting ability of BIOT-NFL to GBM cells. However, it should be noted that at this low concentration of peptide after the dilution of formulation (0.3 μM of BIOT-NFL) the peptide was not present in all fields, and here only confocal images showing the peptide were presented. When cells were treated with BIOT-NFL alone or coupled with B-LNCs (Fig. 7B and C respectively), the peptide was localized in cells, but we did not observe any alteration of the tubulin. Interestingly, the treatment of BIOT-NFL/Col-LNCs (Fig. 7D) shows the effect of Col-LNCs on the destruction of microtubules with the presence of peptide inside cells. Fig. 7E, showed the localisation of BIOT-NFL near to nucleus in cells treated with this coupling (BIOT-NFL/Col-LNCs). Thus, the peptide is suggested to aid the entry of coupled Col-LNCs into GBM cells and then these LNCs exert their activity in cells. Nevertheless, Col-LNCs without the peptide (Col-LNCs) also showed an effect on GBM microtubules (data not shown), thus further investigation or optimization of peptide concentration is still needed.

Based on our first result in this study, the coupling between BIOT-NFL and Col-LNCs may as well provide an additive anti-microtubule effect at appropriate concentrations. However, in these experiments, the concentrations of BIOT-NFL after the dilution of BIOT-NFL/Col-LNCs were very low (ranging from 0.1 to 2.5 μM), and thus the peptide didn't act as an anti-microtubule agent and present the additive effect with Colchicine.

A critical aspect that we faced in this experiment was the low concentrations of BIOT-NFL-peptide following the dilution of formulation. Due to the high toxicity of Colchicine on GBM cells, high dilutions were performed on BIOT-NFL/Col-LNCs, and consequently the resulting concentrations of BIOT-NFL after the dilution were very low ranging from (0.1 μM to 2.5 μM). However, even at these low concentrations, the BIOT-NFL was detected occasionally in GBM cells by immunofluorescence analysis, and an amount of peptide was infrequently found inside cells near the nucleus. When we increased the concentration of peptide coupled with LNCs (more than 0.25 mM of BIOT-NFL), a density of peptide-nanofibers with less adsorbed lipid nanocapsules were observed by microscopic examinations (data not shown). Further, the increasing of peptide concentration has showed a possibility of forming a spontaneous viscous hydrogel at ambient temperature (data not shown) caused by the formation of a very dense network of BIOT-NFL-nanofibers (data demonstrated in our previous work). Therefore, in this study, we could not increase the peptide concentration so as not to change the interaction between peptide-nanofibers and lipid nanocapsules. However, these observations can open the door to a novel pharmaceutical form which might be suitable for a local administration. Thus, in addition to its properties, this peptide might also be used to generate an injectable hydrogel in which the nanocapsules could be dispersed (Drug-loaded LNCs incorporated in BIOT-NFL-nanofibers hydrogel) for possible local chemotherapeutic applications (Bastiancich et al., 2016; Nguyen et al., 2017). Though, the BIOT-NFL concentration still to be optimized in the future work to ensure the use of this peptide as an active targeting agent (active vector that exhibit an anti-tumour activity).

Together with its advantages, GBM-targeting and cell-penetrating ability, anti-microtubule effect, and the formation of long peptide-nanofibers that interact with nanoparticles, BIOT-NFL-peptide may present a possible smart vector for the delivery of different therapeutics to target glioblastoma.

## 5 Conclusion

One of the most challenging issues in the treatment of glioblastoma is the targeted delivery of anti-cancer drugs to the tumour site. The present study established a potential new nanosystem of BIOT-NFL-nanofibers decorated with lipid nanocapsules loaded with an anti-microtubule agent (Colchicine). Peptide-mediated drug delivery system design can facilitate tumour uptake of chemotherapeutic drugs. Colchicine-loaded LNCs were developed in this study and successfully coupled to peptide-nanofibers formed by BIOT-NFL. The coupling with the peptide could facilitate the delivery of coupled Col-LNCs and enhance their anti-tumour activity in GBM cells. The resulting system (nanofibers/nanocapsules) could also be used for the delivery of other therapeutics for possible systemic or local delivery. Such a nanosystem can give rise to more specific accumulation of nanocapsules in tumour and minimize or avoid off-target drug effects. The advantages of the BIOT-NFL-peptide as well as its successful coupling with nanoparticles will likely have important applications in targeted nanotherapy for the treatment of glioblastoma.

## Funding

This work has been supported by 10.13039/501100004099Ligue contre le Cancer 49, Plan Cancer Inserm, and Collège de France-PAUSE program.

## CRediT authorship contribution statement

**H. Alnemeh-Al Ali:** Writing – original draft, Visualization, Software, Investigation, Formal analysis, Data curation, Conceptualization. **J. Bejaud:** Visualization, Supervision, Methodology. **N. Lautram:** Visualization, Validation, Software, Investigation. **A. Dupont:** Visualization, Validation, Software, Investigation. **J. Eyer:** Writing – review & editing, Visualization, Validation, Supervision, Software, Resources, Project administration, Methodology, Investigation, Funding acquisition, Formal analysis, Data curation, Conceptualization.

## Declaration of competing interest

The authors declare no competing interests.

## Data Availability

We have shared the data at the attached file step

## Appendix A Supplementary data

Supplementary material Image 1
