# Supplementary material for: Development of a potential nano-based delivery system combining Colchicine-loaded lipid nanocapsules and BIOT-NFL-peptide to target glioblastoma
Source: Int J Pharm X. 2025 Aug 26;10:100382. doi: 10.1016/j.ijpx.2025.100382 (PMC12418994; doi:10.1016/j.ijpx.2025.100382)
Supplement: Supplementary file 1 — Supplementary material [file mmc1.pdf]

Supplementary data

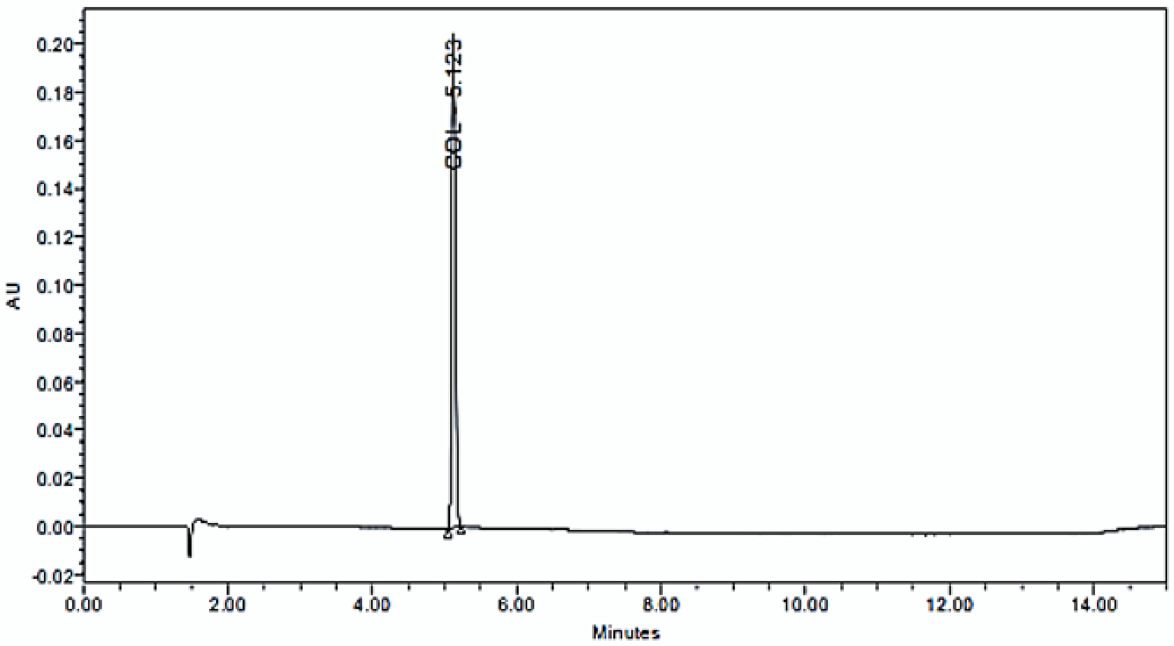

Peak Results

	Name	RT	Area	Height	% Area	Amount	Units	EP s/n
1	COL	5.123	682226	204294	100.00	10.000	µg/mL>	

Figure S1: Detection of Colchicine by Ultra Performance Liquid Chromatography system (UPLC-UV).

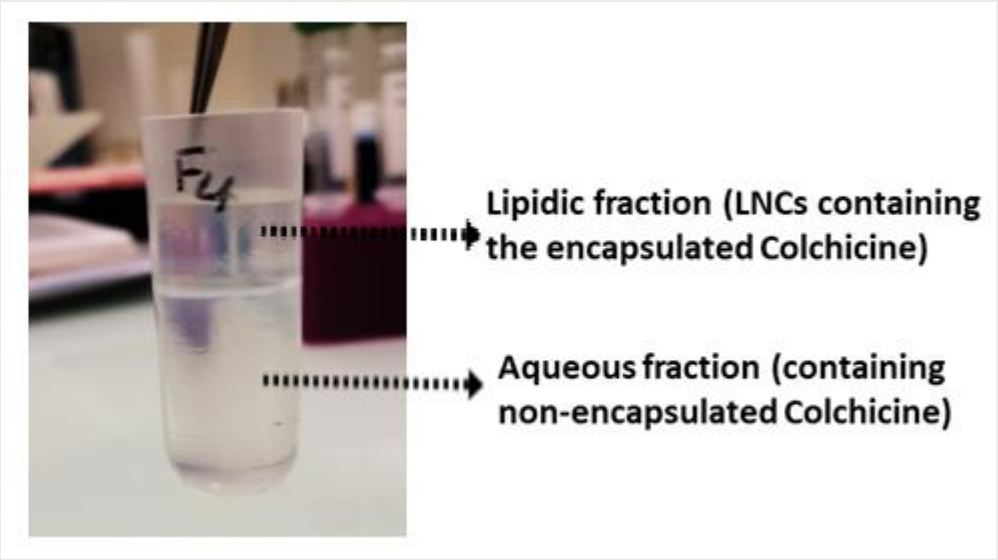

Figure S2: Separation of encapsulated and non-encapsulated Colchicine by ultracentrifugation of Colchicine-loaded lipid nanocapsules.

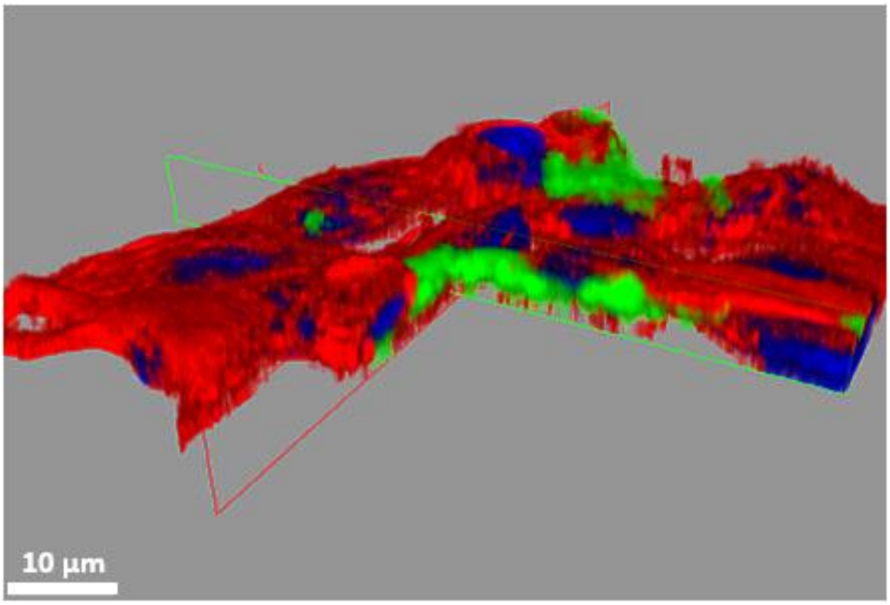

Figure S3: Uptake of the Biotinylated-NFL-peptide (BIOT-NFL) in glioblastoma cells (3D confocal image). 3D confocal microscopy image of rat F98 glioblastoma cells treated with 10 µM of BIOT-NFL for 6 hours. Microtubules were visualized in red, the BIOT-NFL-peptide in green, and nucleus in blue. Scale bars: 10 µm.

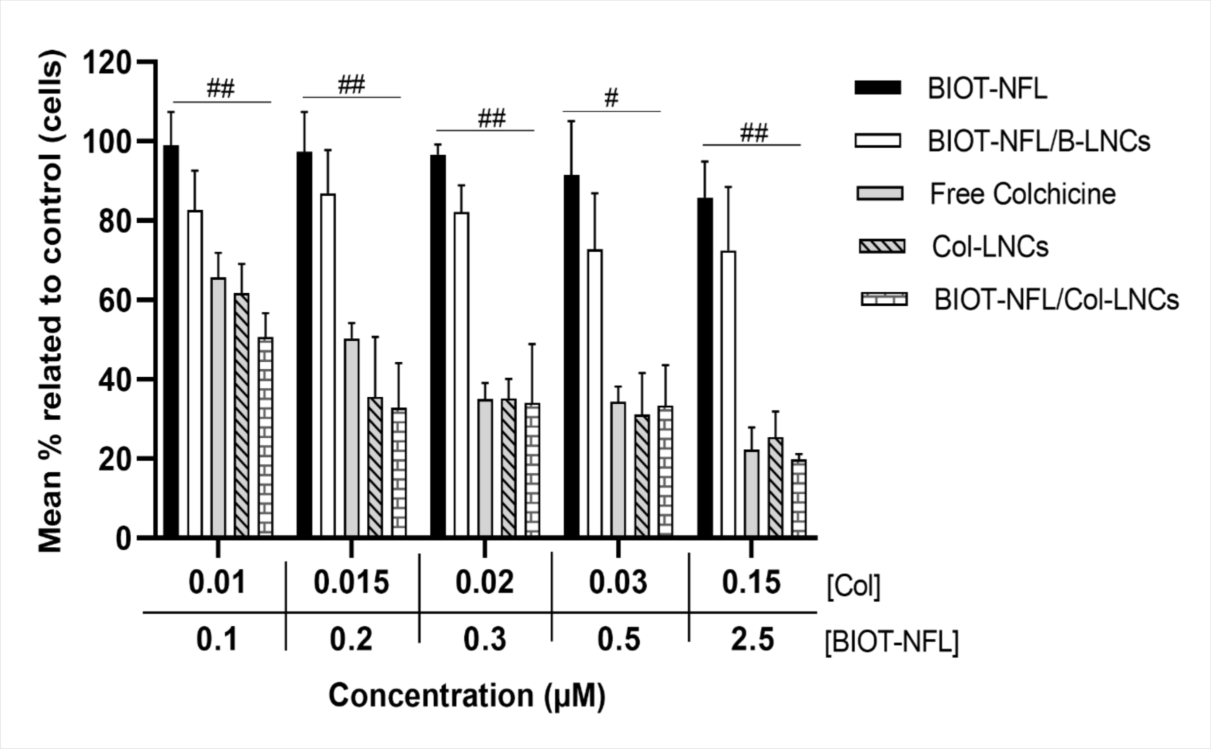

Figure S4: Investigation of the effect of Colchicine lipid nanocapsules coupled or not with BIOT-NFL-peptide on MIA PaCa-2 cells. A MTT assay measured the toxic effect of Col-LNCs (Col-loaded LNCs without the peptide) and BIOT-NFL/Col-LNCs (Col-loaded LNCs coupled with BIOT-NFL) on MIA PaCa-2 cells after 72 hours incubation. Cells were treated with free-Col, free-BIOT-NFL and BIOT-NFL/B-LNCs (blank-LNCs coupled with BIOT-NFL) at equivalent concentrations. Values are expressed as mean ± SEM, (n = 3). ($^{\#}$); BIOT-NFL/Col-LNCs as compared with BIOT-NFL/B-LNCs.

($^{\#}p < 0.05$; $^{\#\#}p < 0.005$ and $^{\#\#\#}p < 0.001$).

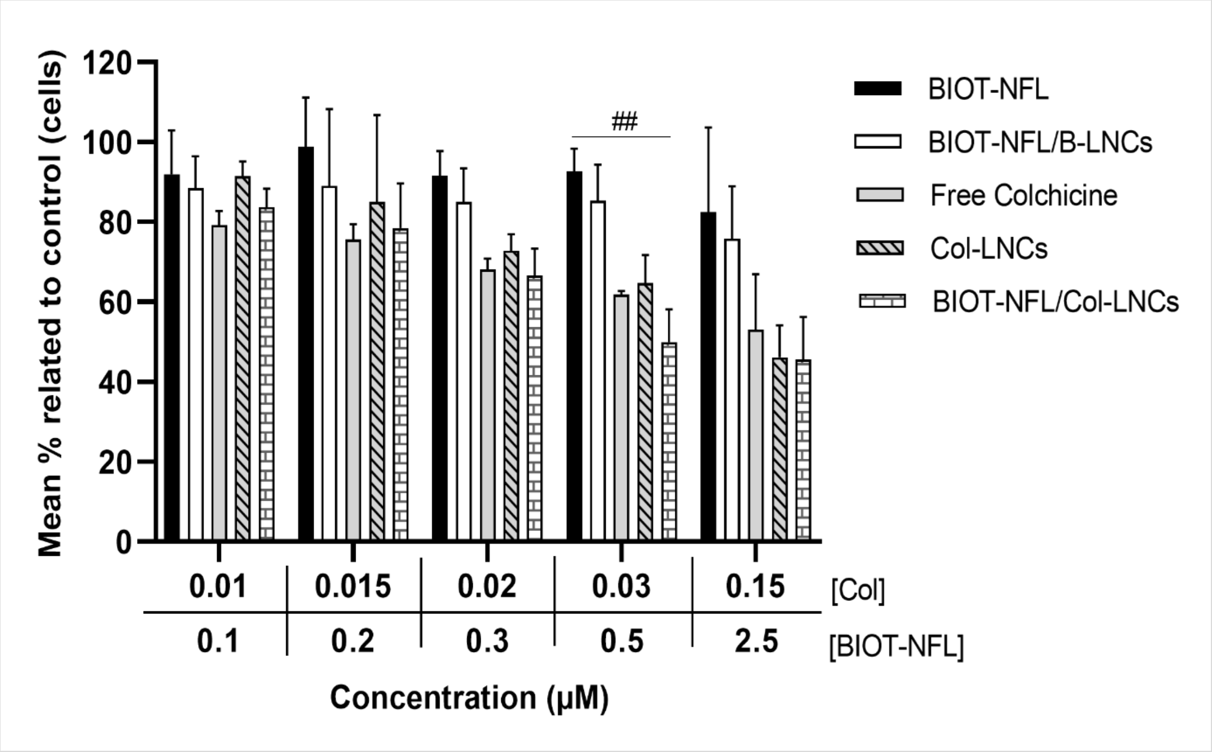

Figure S5: Investigation of the effect of Colchicine lipid nanocapsules coupled or not with BIOT-NFL-peptide on SH-SY5Y cells. A MTT assay measured the toxic effect of Col-LNCs (Col-loaded LNCs without the peptide) and BIOT-NFL/Col-LNCs (Col-loaded LNCs coupled with BIOT-NFL) on SH-SY5Y cells after 72 hours incubation. Cells were treated with free-Col, free-BIOT-NFL and BIOT-NFL/B-LNCs (blank-LNCs coupled with BIOT-NFL) at equivalent concentrations. Values are expressed as mean ± SEM, (n = 3). ($^{\#}$); BIOT-NFL/Col-LNCs as compared with BIOT-NFL/B-LNCs.

($^{\#}p < 0.05$; $^{\#\#}p < 0.005$ and $^{\#\#\#}p < 0.001$).
